# Impact of a ban on the open display of tobacco products in retail outlets on never smoking youth in the UK: findings from a repeat cross-sectional survey before, during and after implementation

**DOI:** 10.1136/tobaccocontrol-2018-054831

**Published:** 2019-05-14

**Authors:** Allison Ford, Anne Marie MacKintosh, Crawford Moodie, Mirte A G Kuipers, Gerard B Hastings, Linda Bauld

**Affiliations:** 1 Institute for Social Marketing, University of Stirling, Stirling, UK; 2 UK Centre for Tobacco and Alcohol Studies, University of Stirling, Stirling, UK; 3 Department of Public Health, Academic Medical Center, University of Amsterdam, Amsterdam, The Netherlands; 4 Usher Institute, College of Medicine and Veterinary Medicine, University of Edinburgh, Edinburgh, UK

**Keywords:** advertising and promotion, prevention, public policy

## Abstract

**Background:**

In the UK, a ban on the open display of tobacco products at the point of sale (POS) was phased in between 2012 and 2015. We explored any impact of the ban on youth before, during and after implementation.

**Methods:**

A repeat cross-sectional in-home survey with young people aged 11–16 years old in the UK was conducted preban (2011, n=1373), mid-ban (2014, n=1205) and postban (2016, n=1213). The analysis focuses on the never-smokers in the sample (n=2953 in total). Preban, we quantified the associations of noticing cigarettes displayed at POS and cigarette brand awareness with smoking susceptibility. We measured any change in noticing cigarettes displayed at POS, cigarette brand awareness and smoking susceptibility between preban, mid-ban and postban. Postban, we assessed support for a display ban, perceived appeal of cigarettes and perceived acceptability of smoking as a result of closed displays.

**Results:**

Preban, noticing cigarettes displayed at POS (adjusted OR [AOR]=1.97, 95% CI 1.30 to 2.98) and higher brand awareness (AOR=1.15, 95% CI 1.03 to 1.29) were positively associated with smoking susceptibility. The mean number of brands recalled declined from 0.97 preban to 0.69 postban (p<0.001). Smoking susceptibility decreased from 28% preban to 23% mid-ban and 18% postban (p for trend <0.001). Postban, 90% of never-smokers supported the display ban and indicated that it made cigarettes seem unappealing (77%) and made smoking seem unacceptable (87%).

**Conclusions:**

Both partial and full implementation of a display ban were followed by a reduction in smoking susceptibility among adolescents, which may be driven by decreases in brand awareness.

## Introduction

In countries that have introduced comprehensive bans on tobacco advertising, promotion and sponsorship, the retail environment becomes more important for tobacco companies as the display of tobacco products at the point of sale (POS) allows them to showcase their products.^1^ To make the most of this opportunity, they invest heavily in ensuring that tobacco brands are attractively packaged and prominently positioned.^2 3^ A systematic review and meta-analysis found a consistent positive association between POS tobacco promotion or displays and increased smoking and smoking susceptibility among children and adolescents.^4 5^ Open tobacco displays also evoke positive attitudes among young people, including the perception that displays are ‘cool’ and attract people to smoke.^6^ As such, requiring tobacco products to be kept out of sight in shops may help to protect youth.^7^ However, there is limited research exploring the impacts of a ban on the open display of tobacco products, hereafter referred to as a ‘display ban’, on youth.

A display ban was first introduced by Iceland in 2001, with 20 countries having implemented a ban by 2016.^8^ In the UK a display ban was not introduced for all retailers at the same time. Instead, it was first introduced in large shops (over 280 m^2^ of retail space) in England in April 2012, in Northern Ireland in October 2012, in Wales in December 2012, and in Scotland in April 2013. The difference in timing between the four countries that comprise the UK was because each was free to implement the display ban when they chose, and in the case of Scotland a legal challenge delayed its introduction. The full ban, covering small shops as well, came into force in April 2015 throughout the UK.^9^ The UK ban stipulates that the tobacco gantry or storage unit must be fully covered to obscure the view of tobacco products completely. Although the rules do not stipulate how units should be covered, most retailers use sliding doors or hanging covers, although curtains are also allowed. When retrieving products the area which can be displayed must not exceed 1.5 m^2^.

Evidence on the impact of display bans suggests that they may help to contribute to a reduction in adolescent smoking rates. A study found that across six European countries which had implemented a display ban, this measure was associated with a 15% decrease in the odds of adolescent regular smoking.^10^ Evidence from Australia^11^ and New Zealand^12^ also found a reduction in adolescent smoking rates, but not in Ireland.^13^ However, conducting repeat interviews with a small sample over a short time period may have contributed to the null finding.

Some studies have explored potential mechanisms through which display bans may reduce smoking prevalence. After implementation of a display ban, approximately 40% of a sample of adolescents in Ireland thought that the removal of tobacco from view in shops made it easier for children not to smoke.^13^ Adolescents in Norway^14^ and Australia^11^ recognised fewer tobacco brands after a display ban, and young people in Ireland^13^ and Australia^11^ were less likely to overestimate the smoking prevalence among their peers. A study in England found an association between a display ban and a lower incidence of adolescent regular smokers (ages 11–15) purchasing cigarettes from shops, but no changes in perceived difficulty in purchasing cigarettes from shops.^15^ Another English study, conducted with adolescents (ages 11–16) in Nottingham before the full ban came into force, found that a partial display ban did not result in reduced susceptibility to smoke.^16^

We build on past research by exploring the impacts of a display ban, in a country where it has been phased in over a period of several years, among a UK-wide sample (England, Scotland, Wales and Northern Ireland) of young people aged 11-16 years old before, during and after implementation. Specifically, we aimed to address three research questions: (1) What are the preban associations of noticing cigarettes displayed at POS and cigarette brand awareness with smoking susceptibility? (2) To what extent did noticing cigarettes displayed at POS, cigarette brand awareness and smoking susceptibility change between preban, mid-ban and postban measurements? (3) What were the mid-ban and postban levels of support for the display ban, and did having cigarettes behind closed shutters make cigarettes seem unappealing and smoking seem unacceptable?

## Methods

### Study design

Data were from waves 6, 7 and 8 of the Youth Tobacco Policy Survey, a long-term, repeat cross-sectional study that examines the impacts of tobacco control policies in the UK on young people. Wave 6 was conducted in August–September 2011, before the display ban was introduced. Wave 7 was conducted in August–September 2014, after the ban had been introduced in large shops but prior to it being introduced in smaller shops. Wave 8 was conducted in August–September 2016, 16–17 months after the ban had been fully implemented. A market research company was commissioned to recruit participants, secure participant and parental consent prior to each interview, and conduct the fieldwork. The fieldwork comprised in-home, face-to-face interviews, accompanied by a self-administered questionnaire to gather more sensitive information on smoking behaviour and susceptibility to smoke.

### Sample

The sample comprised young people aged 11–16 years (2011: n=1373; 2014: n=1205; 2016: n=1213) drawn from households across the UK, using random location quota sampling. Sampling involved random selection of 92 electoral wards, stratified by Government Office Region and A Classification Of Residential Neighbourhoods classification (a geodemographic classification system that describes demographic and lifestyle profiles of small geographical areas) to ensure coverage of a range of geographical areas and sociodemographic backgrounds. For more information on the sampling and methodology, see elsewhere.^17–19^

### Measures

#### Sociodemographic characteristics

At each wave age, gender and smoking by parents, siblings (if any) and close friends were obtained. Social grade was determined by the occupation of the chief income earner in the household (ABC1=middle class, C2DE=working class). These groupings are based on the widely used UK demographic classifications system derived from the National Readership Survey. Middle class (ABC1) reflects managerial, administrative and professional occupations. Working class (C2DE) reflects skilled and unskilled manual workers, and casual or lowest grade workers.

#### Cigarette brand awareness

A single item asked participants to name cigarette brands they had heard of. No prompts were given and a maximum of 10 brands were recorded.

#### Smoking susceptibility

Never-smokers were those who indicated that they had never tried or experimented with smoking, not even a few puffs. Susceptibility, defined as the absence of a firm decision not to smoke,^20^ was measured across three items addressing the likelihood that they would (1) be smoking when they are 18, (2) smoke a cigarette at any time during the next year and (3) smoke if one of their friends offered them a cigarette. Response options for each were ‘definitely not’, ‘probably not’, ‘probably yes’ and ‘definitely yes’. Never-smokers were classed as non-susceptible if they responded ‘definitely not’ for all three items, and as susceptible if their response was anything other than ‘definitely not’ to any of the three items.

#### Noticing displays

This was measured via a single item. In 2011, preban, participants were asked: ‘In the last month, have you seen cigarette packets being displayed, including on shelves or on the counter?’ In the mid-ban and postban survey waves, the item was reworded to make it clear to participants that they were being asked about the open display of cigarette packets: ‘In the last month, have you seen cigarette packets being openly displayed, including on shelves or on the counter? By openly displayed, I mean without any shutters or screens covering the packs’. To each question participants could respond ‘yes’, ‘no’ or ‘don’t know’. Those who answered ‘yes’ were classed as having ‘noticed’ displays. Given the lack of positive affirmation on noticing, those who answered ‘don’t know’ were combined with the ‘no’ responses and classed as having ‘not noticed’.

#### Perceptions of, and support for, closed displays

Three items assessed perceptions of closed displays mid-ban and postban: (1) support for cigarettes being out of sight; (2) perceived appeal of cigarettes; and (3) perceived acceptability of smoking resulting from closed displays. Participants were asked: ‘Now I’d like to find out what you think about cigarette packets being hidden behind shutters or screens in shops. Can you read the statements on both sides of this card and give me the number that best describes what you think?’ For each item responses were measured on a 5-point scale: (1) shops should have to keep cigarette packs behind closed shutters (1)/shops should be allowed to have cigarette packs visible (5); (2) having cigarette packs behind shutters in shops makes cigarettes seem unappealing (1) or appealing (5); and (3) having cigarette packs behind shutters in shops makes me think that it’s NOT OK to smoke (1) or OK to smoke (5).

### Statistical analysis

Data were analysed using SPSS V.23. Descriptive data and bivariate analysis were weighted to standardise by age and gender across survey years. The analysis focused on the 2953 never-smokers (2011: n=1025; 2014: n=948; 2016: n=980). First, we analysed the 2011 data. Associations between preban measures of susceptibility to smoke and noticing cigarette packets at POS and brand recall were examined using logistic regression to enable the potential influence of demographic and smoking-related variables to be controlled. The control variables were sibling, close friend and parental smoking; age; gender; and social grade.

Second, we analysed changes over time. Bivariate analysis was conducted using the χ^2^ test to examine potential differences, across survey years, in (1) noticing of cigarette packets at POS and (2) susceptibility to smoke. One-way analysis of variance (ANOVA) was conducted to test for differences in the number of brands recalled across survey years. Within the ANOVA, the Welch’s *F* test was used, as homogeneity of variance assumption was not met for the brand recall data. Additionally, logistic regression was conducted to test for differences across survey years in susceptibility after controlling for smoking-related and demographic variables. The same control variables used to model the 2011 data were included, with survey year added. The mid-ban survey year (2014) was set as the reference category to explore whether susceptibility changed between preban and mid-ban measures and between mid-ban and postban measures. All logistic regressions were conducted on unweighted data as age, gender and social grade were control variables. Age was entered as a categorical variable as the assumptions of linearity of the logit were not met for all analyses. For the categorical age variable, the ‘difference’ contrast, within SPSS logistic regression, was used to test the influence of each increasing age group relative to all younger ages (eg, 12 vs 11, 13 vs 11–12, 14 vs 11–13). In each logistic regression cases were excluded if they had missing data on the dependent variable or any of the independent variables.

Third, we describe mid-ban and postban percentages of support for the display ban, perceived appeal of cigarettes and perceived acceptability of smoking as a result of closed displays. For each item, the percentage who answered codes ‘1’ or ‘2’ (indicating support) on the 5-point scale is reported. For each item, those who did not answer (ie, did not provide a score between ‘1’ and ‘5’) were treated as missing data.

## Results

### Sample description

After excluding cases that had missing information for smoking status (n=33), 78% (n=2920) of the weighted sample were never-smokers (table 1). The mean age of never-smokers was 13.17 years (SD=1.64), with 50% (n=1473) male and 57% (n=1648) working class (C2DE). Approximately a quarter, 23% (n=673), were categorised as susceptible to smoke.

**Table 1:** Sample profile based on unweighted and weighted frequencies

	Full sample				Never-smokers			
	Unweighted		Weighted*		Unweighted		Weighted*	
	n	%	n	%	n	%	n	%
Total	3791	100	3788	100	2953	100	2920	100
Gender								
Male	1898	50	1895	50	1502	51	1473	50
Female	1893	50	1892	50	1451	49	1448	50
Age								
11	719	19	627	17	682	23	596	20
12	596	16	626	17	548	19	576	20
13	643	17	639	17	537	18	534	18
14	658	17	624	16	513	17	487	17
15	621	16	640	17	396	13	411	14
16	551	15	630	17	277	9	316	11
Mean (SD)	13.40 (1.70)		13.51 (1.71)		13.08 (1.63)		13.17 (1.64)	
Social grade								
Middle class (ABC1)	1529	41	1530	41	1258	43	1247	43
Working class (C2DE)	2230	59	2226	59	1669	57	1648	57
Not specified†	32		32		26		26	
Smoking status								
Never smoked	2953	79	2920	78	–	–	–	–
Ever smoked	804	21	834	22	–	–	–	–
Not specified†	34		33					
Susceptibility								
Non-susceptible	–	–	–	–	2258	77	2235	77
Susceptible	–	–	–	–	683	23	673	23
Not specified†					34		33	
Year								
2011	1373	36	1372	36	1025	35	1016	35
2014	1205	32	1203	32	948	32	948	32
2016	1213	32	1213	32	980	33	980	33

*Data are weighted to standardise by age and gender.

†Cases excluded due to missing data.

### Preban

In 2011, 28% of never-smokers (n=282) were categorised as susceptible. Most never-smokers (81%, n=820) noticed cigarette packets displayed at POS, with the mean number of brands recalled less than one (0.97, SD=1.26). Logistic regression, after controlling for smoking-related and demographic variables, found noticing cigarette packets openly displayed preban to be positively associated with susceptibility (adjusted OR [AOR]=1.97, 95% CI 1.30 to 2.98; table 2). Additionally, brand recall was also found to have a modest positive association with susceptibility after adjusting for all other variables (AOR=1.15, 95% CI 1.03 to 1.29).

**Table 2:** Logistic regression of preban (2011) association between never-smokers’ susceptibility to smoke and noticing cigarettes openly displayed and brand awareness, controlling for smoking-related and demographic variables

	Never-smokers’ susceptibility to smoke 1=susceptible (n=282), 0=not (n=719)				
	n	AOR*	95% CI Lower	95% CI Upper	P value
Any close friends smoke					
No	697	Ref			
Yes	304	2.05	1.48	2.84	<0.001
Any siblings smoke					<0.001
No/No siblings	836	Ref			
Yes	137	2.31	1.54	3.47	<0.001
Not sure/not stated	28	2.19	0.99	4.86	0.053
Parents smoke					0.283
Neither smokes	567	Ref			
Either	344	1.14	0.82	1.59	0.436
Not sure/not stated	90	1.48	0.90	2.44	0.121
Gender					
Male	515	Ref			
Female	486	0.93	0.69	1.24	0.617
Age					0.026
11	209	Ref			
12 *vs 11*	200	1.38	0.85	2.24	0.187
13 *vs 11–12*	199	1.45	0.98	2.15	0.061
14 *vs 11–13*	171	1.11	0.75	1.65	0.591
15 *vs 11–14*	130	1.19	0.78	1.82	0.421
16 *vs 11–15*	92	0.49	0.28	0.85	0.011
Social grade					
Middle class (ABC1)	461	Ref			
Working class (C2DE)	540	0.78	0.58	1.06	0.112
Notice openly displayed					
No or don’t know	195	Ref			
Yes	806	1.97	1.30	2.98	0.001
Brand awareness	1001	1.15	1.03	1.29	0.015
	Model χ²= 86.530, df=14, p<0.001. Hosmer and Lemeshow χ²= 10.761, df=8, p=0.216. Cases correctly classified: 74%. 24 cases excluded due to missing data on one or more independent variables.				

*Adjusted for all other variables in the model.

AOR, adjusted OR; ref, reference category.

### Changes in noticing cigarette packs, brand recall and susceptibility from 2011 to 2016

Table 3 shows how the proportion who noticed cigarette packs at POS, the mean number of cigarette/tobacco brands recalled and the prevalence of susceptibility changed from preban to mid-ban, to postban. Bivariate analyses indicated a decrease in noticing cigarette packets from 81% preban to 28% postban (p<0.001), a decrease in average brand recall from 0.97 (SD=1.26) to 0.69 (SD=1.09) (p<0.001), and a reduction in susceptibility from 28% preban to 18% postban (p<0.001).

**Table 3:** Noticing displays, brand awareness and susceptibility, preban, mid-ban and postban of display

Base: all never-smokers (weighted)	Preban (2011)		Mid-ban (2014)		Postban (2016)		P value differences by year
	n	%	n	%	n	%	
% Noticing openly displayed at point of sale	820	81	613	66	271	28	<0.001*
% of never-smokers susceptible to smoke	282^a^	28	215^b^	23	177	18	<0.001*
Mean (SD) number of brands recalled	0.97 (1.26)		0.86 (1.28)		0.69 (1.09)		<0.001†

Number of cases excluded due to missing data: ^a^n=6; ^b^n=6.

*χ^2^ test for trend.

†One-way analysis of variance Welch’s *F.*

Logistic regression, controlling for smoking-related and demographic variables, indicated that the odds of never-smokers noticing cigarette packets at POS decreased following the display ban (table 4). Preban, the odds of never-smokers noticing cigarette packets at POS were more than twice as high compared with mid-ban (AOR=2.13, 95% CI 1.73 to 2.63). The odds of noticing cigarette packets at POS reduced further postban (AOR=0.20, 95% CI 0.16 to 0.24).

**Table 4:** Logistic regression of association between noticing cigarettes displayed and susceptibility, and survey stage, controlling for smoking-related and demographic variables

	Never-smokers noticing openly displayed at point of sale 1=notice (n=1693), 0=not (n=1225)					Never-smokers’ susceptibility to smoke 1=susceptible (n=673), 0=not (n=2239)				
	n	AOR*	95% CI Lower	95% CI Upper	P value	n	AOR*	95% CI Lower	95% CI Upper	P value
Survey stage					<0.001					<0.001
Preban (2011)	1001	2.13	1.73	2.63	<0.001	1 001	1.31	1.06	1.62	0.014
Mid-ban (2014)	937	Ref				931	Ref			
Postban (2016)	980	0.20	0.16	0.24	<0.001	980	0.79	0.63	0.99	0.040
Any close friends smoke										
No	2181	Ref				2176	Ref			
Yes	737	1.39	1.13	1.72	0.002	736	2.21	1.81	2.71	<0.001
Any siblings smoke					0.532					<0.001
No/No siblings	2425	Ref				2424	Ref			
Yes	381	1.15	0.89	1.49	0.274	380	1.62	1.26	2.07	<0.001
Not sure/not stated	112	1.08	0.70	1.68	0.721	108	2.18	1.43	3.31	<0.001
Parents smoke					0.017					<0.001
Neither smokes	1641	Ref				1641	Ref			
Either	944	1.32	1.08	1.60	0.005	942	1.31	1.07	1.60	0.010
Not sure/not stated	333	1.22	0.92	1.60	0.167	329	1.72	1.30	2.28	<0.001
Gender										
Male	1484	Ref				1483	Ref			
Female	1434	0.86	0.73	1.02	0.084	1429	0.84	0.70	1.01	0.058
Age					0.207					0.002
11	673	Ref				672	Ref			
12 *vs 11*	543	1.11	0.86	1.44	0.418	541	1.06	0.79	1.42	0.704
13 *vs 11–12*	528	1.21	0.96	1.54	0.111	528	1.42	1.11	1.81	0.006
14 *vs 11–13*	506	1.09	0.87	1.38.	0.446	504	1.14	0.90	1.45	0.284
15 *vs 11–14*	393	1.16	0.90	1.49	0.239	393	1.05	0.81	1.36	0.723
16 *vs 11–15*	275	0.82	0.62	1.10	0.195	274	0.61	0.44	0.84	0.003
Social grade										
Middle class (ABC1)	1257	Ref				1255	Ref			
Working class (C2DE)	1661	1.00	0.84	1.18	0.955	1657	0.96	0.80	1.16	0.684
	Model χ²=656.03, df=14, p<0.001. Hosmer and Lemeshow χ²=11.742, df=8, p=0.163. Cases correctly classified: 73%. 35 cases excluded due to missing data on one or more independent variables.					Model χ²=170.52, df=14, p<0.001. Hosmer and Lemeshow χ²=4.471, df=8, p=0.812. Cases correctly classified: 77%. 41 cases excluded due to missing data on one or more independent variables.				

*Adjusted for all other variables in the model.

AOR, adjusted OR; ref, reference category.

Logistic regression, controlling for smoking-related and demographic variables, indicated that susceptibility decreased following the display ban (table 4). Never-smokers in 2011 (preban) had higher odds than never-smokers in 2014 (mid-ban) of being susceptible (AOR=1.31, 95% CI 1.06 to 1.62), with never-smokers in 2016 (postban) having lower odds of being susceptible than never-smokers in 2014 (AOR=0.79, 95% CI 0.63 to 0.99).

### Perceptions of, and support for, closed displays

Most never-smokers at mid-ban (86%, n=783) and postban (90%, n=841) indicated that ‘Shops should have to keep cigarette packs behind closed shutters’ (table 5). Around three-quarters at mid-ban (73%, n=673) and postban (77%, n=688) held the view that ‘Having cigarette packs behind shutters in shops makes cigarettes seem unappealing’, and over four-fifths at mid-ban (83%, n=764) and postban (87%, n=790) considered that ‘Having cigarette packs behind shutters in shops makes me think that it’s NOT OK to smoke’.

**Table 5:** Perceptions of and support for closed displays, mid-ban and postban

Base: all never-smokers (weighted)	Mid-ban (2014)		Postban (2016)	
	n	%	n	%
% of never-smokers who thought*:				
Shops should have to keep cigarette packs behind closed shutters.	783†	86	841‡	90
Having cigarette packs behind shutters in shops makes cigarettes seem unappealing.	673§	73	688¶	77
Having cigarette packs behind shutters in shops makes me think that it’s NOT OK to smoke.	764**	83	790††	87

*Proportion answering either code ‘1’ or ‘2’ (indicating support) to each item. Number of cases excluded due to missing values: †n=26, ‡n=40, §n=19, ¶n=82, **n=16, ††n=58.

## Discussion

### Main findings

This study examined the responses of never smoking youth to a POS display ban for tobacco products in the UK. The main findings are that (1) preban, noticing cigarettes displayed at POS and higher brand awareness were positively associated with smoking susceptibility; (2) mid-ban, there was a significant reduction in susceptibility; (3) mid-ban and postban, most young never-smokers were supportive of a display ban; (4) mid-ban and postban, most young never-smokers perceived that the display ban made cigarettes seem unappealing and smoking seem unacceptable; and (5) postban, there were reductions in brand awareness and further reductions in susceptibility.

### Interpretation of results

In the UK smoking prevalence in young people (ages 11–16) had declined significantly in the years preceding the initial wave of this study. Nonetheless, prior to the display ban, we found that a large minority of young never-smokers remained susceptible to smoking. Therefore, preban, the power of the open display of tobacco products is evident, consistent with other studies which have shown associations between POS displays of tobacco and youth susceptibility.^4 5^ This finding suggests that either noticing cigarettes at POS and higher brand awareness influences susceptibility, or an already present susceptibility influences the noticing of cigarettes and brand awareness. Both directions of association give cause for concern, suggesting that open displays either influence future smoking or that young never-smokers use the display of tobacco products to shape or reinforce their smoking decisions. A display ban is therefore a potential protective factor against any vulnerability to tobacco displays for young never-smokers, who have no existing involvement with the product. As the display ban was phased in, we found a significant decrease in susceptibility from preban to mid-ban, to postban. This is in contrast to results from a study conducted in England which found no reduction in susceptibility; however, that study only covered the period before the full ban came into force.^16^ The decline in susceptibility reported in our study is likely the cumulative effect of the myriad tobacco control policies introduced in the UK over the last two decades—especially comprehensive controls on other forms of tobacco marketing, for example, the Tobacco Advertising and Promotion Act (TAPA)—rather than the display ban in isolation. Nonetheless, our study suggests that the display ban has been an important component of the UK’s tobacco control strategy, which has delivered historically low levels of youth smoking.^21^ It was only after the introduction of the display ban that the Youth Tobacco Policy Survey observed the first reduction in smoking susceptibility. Preban this measure had remained constant, with 27% classified as susceptible in 2008^22^ and 28% in 2011.

The study also points to the power of the brand in recruiting young smokers. Brand awareness and the associated brand imagery is the culmination of the marketing effort and a key promotional driver of consumption, particularly among youth, who are considered most vulnerable.^23^ The lack of familiarity with cigarette brands in 2011, when the mean number of brands recalled was less than one, 0.97, is likely the result of the TAPA, a comprehensive ban on tobacco advertising, promotion and sponsorship implemented between 2003 and 2005. However, brand recall dropped significantly, to 0.69 postban. Protecting young people from the persuasive power of the brand is an important way of preventing youth smoking. A key strength of a display ban is that it enhances this protection by reducing exposure to the brand.

Our study also highlights the importance of a comprehensive approach to tobacco control. Although a partial ban—as measured at the midpoint of our study when only larger shops had been required to implement the ban—had some benefits, it was only with full implementation in all shops that the policy became fully effective. It is possible that the reductions in susceptibility and brand awareness after full implementation may be due to lagged effects of the ban first implemented in larger shops, or the cumulative effect of the ban in both larger and smaller shops, providing a useful reminder that small shops, such as newsagents and garages, are also important players in youth smoking prevention.^15^

Both mid-ban and postban, young people’s attitudes provide additional support for tobacco display bans. While young people perceive open displays as ‘cool’ and attracting people to smoke,^6^ this study shows that display bans communicate the message that cigarettes are not an appealing product and that it is not okay to smoke. That 90% of our postban sample supported closed displays gives the policy a powerful endorsement; it may also be indicative of the denormalisation of tobacco use and the general trend towards more negative attitudes to smoking in the UK.^24^

Finally, an apparent quirk in the data is worth discussing. A relatively high proportion of our sample (28%) still reported seeing packs at POS postban, consistent with other studies.^12 15^ Two explanations can be posited for this. First, as high levels of retailer compliance have been found with display bans, at least in high-income countries,^12 25–28^ it is possible that participants retain residual memory of displays preban. Alternatively, and perhaps more likely, even with a full display ban, there will be incidental exposure, where children see cigarettes when the shutters are opened, whether to serve tobacco to other customers or for restocking.

### Strengths and limitations

This study extends other work that found an effect of POS display bans on youth smoking rates^10–12^ by also demonstrating a reduction in youth smoking susceptibility among never-smokers during and after implementation. While susceptibility cannot tell us that young never-smokers will definitely go on to smoke, it is a well-validated measure of future smoking intent.^20^ Although the cross-sectional study design cannot demonstrate causality, it nevertheless provides evidence of population-level changes in relevant outcomes during the multiple phases of implementation across the UK. It is likely, however, that other tobacco marketing controls, in addition to policies such as enhanced health warning labels, smoke-free legislation and increased tobacco taxation, may have contributed to these changes. Although the sample age limit is 16, display bans may have also affected older teens and young adults, for example, with declines in smoking rates seen among those aged 18–24 years old in England.^29^ It is possible that the survey administration method may have resulted in social desirability bias. Interviews for all waves were conducted in-home, where a family member may have been present, and participants may have been worried about showing positive perceptions about tobacco. The face-to-face survey attempts to protect participants’ privacy, and limit desirability bias, through the use of showcards which enable participants to read responses from the card and give the number which corresponds to their answer. This makes it difficult for anyone else in the room to follow what response has been given. Finally, the exclusion of rural areas and the reliance on non-probability sampling methods for the final stage of sample selection mean that this sample cannot be said to be completely representative of, or absolutely predictive for, all adolescents in the UK. However, the sample is large enough to be strongly indicative of the complete UK adolescent population, and it is useful to examine results from the sample to gain insight into likely patterns of associations in the population of adolescents in relation to susceptibility to smoke.

## Conclusions

Both partial and full implementation of a display ban in the UK were followed by a reduction in smoking susceptibility among adolescents. This may be related to the decrease in brand awareness which occurred alongside implementation of the display ban. This suggests that placing tobacco out of sight helps safeguard young people and justifies this policy approach in the UK and elsewhere. It will be important to continue to monitor these measures over time, particularly to see any further impacts alongside the full implementation of standardised packaging in the UK in May 2017, which further restricts the ability of tobacco companies to advertise their brands and communicate positive messages to youth.

**What this paper adds**

**What is already known on this subject**

- In countries that have introduced tobacco advertising and promotion bans, showcasing tobacco products at the point of sale (POS) has become more important for tobacco companies.
- Studies have shown positive associations between POS displays and increased smoking, smoking susceptibility and positive attitudes among youth.

**What important gaps in knowledge exist on this topic**

- Evidence on the impact of a display ban on youth before, during and after implementation is lacking.
- There is limited knowledge on youth support for a display ban, and perceived appeal of cigarettes and acceptability of smoking as a result of closed displays.

**What this paper adds**

- Preban, noticing cigarettes displayed at POS and higher brand awareness were associated with smoking susceptibility.
- Implementation of a display ban was followed by reductions in smoking susceptibility and cigarette brand awareness among youth.
- Young never-smokers’ support for a display ban was very high mid-ban and postban, and closed displays were perceived to make cigarettes seem unappealing and smoking seem unacceptable.
